# Application of Fractional Calculus to Modeling the Non-Linear Behaviors of Ferroelectric Polymer Composites: Viscoelasticity and Dielectricity

**DOI:** 10.3390/membranes11060409

**Published:** 2021-05-29

**Authors:** Ruifan Meng

**Affiliations:** Institute for Systems Rheology, School of Mechanical and Electrical Engineering, Guangzhou University, Guangzhou 510006, China; rfmeng@gzhu.edu.cn

**Keywords:** ferroelectric polymer composites, fractional order calculus, viscoelasticity, dielectricity

## Abstract

Ferroelectric polymer composites normally show non-linear mechanical and electrical behaviors due to the viscoelastic and dielectric relaxation of polymer matrixes. In this paper, a fractional calculus approach is used to describe the non-linear behavior of ferroelectric polymer composites from both viscoelastic and dielectric perspectives. The fractional elements for viscoelasticity and dielectricity are “spring-pot” and “cap-resistor”, which can capture the intermediate properties between spring and dashpot or capacitor and resistor, respectively. For modeling the viscoelastic deformation, the “spring-pot” equation is directly used as the fractional mechanical model. By contrast, for the dielectricity of ferroelectric polymer composites, which is usually characterized by dielectric constants and dielectric losses, the “cap-resistor” equation is further formulated into the frequency domain by Fourier transform to obtain the fractional order dielectric model. The comparisons with experimental results suggest that the proposed models can well describe the viscoelastic deformation as well as the frequency dependence of the dielectric constant and dielectric loss of ferroelectric polymer composites. It is noted that the fractional order dielectric model needs to be separated into two regions at low and high frequencies due to the polarization effect. Additionally, when the dipole relaxations occur at higher frequencies, the proposed model cannot describe the rise of the dielectric loss curve.

## 1. Introduction

Nowadays, ferroelectric polymer composites have been considered as a substitute for inorganic ferroelectrics as well as for ferroelectric polymers [1]. Inorganic ferroelectrics are usually heavy, brittle, toxic, and require high temperature processing, while polymer-based ferroelectrics suffer from some drawbacks, such as low spontaneous polarization and slow switching time. By composite technology, the mechanical, ferroelectric, and other properties of ferroelectric polymer composites could be enhanced or modified to meet diverse functional aspects of technological applications.

The most studied and widely used ferroelectric polymers are polyvinylidene fluoride (PVDF) and its copolymers with trifluorethylene P (VDF-TrFE) and hexafluoropropylene (PVDF-HFP) [2]. The fillers of ferroelectric polymer composites are usually inorganic particles with high dielectric constants such as BaTiO_3_, TiO_2_, and PZT (PbZr_x_Ti_1-x_O_3_) [3]. By introducing the fillers into a ferroelectric polymer matrix, the resulting composite materials will exhibit multiple-phase properties. They have the features of polymers, such as flexibility, softness, light weight, and low thermal conductivity, along with the ferroelectric properties that are widely used in sensors, actuators, transducers, and other electromechanical devices [4]. Among these features, the non-linear behaviors of ferroelectric polymer composites can be concluded as the mechanical manifestation of viscoelasticity and the electrical manifestation of dielectricity, which are of great concern in applications [5]. In terms of viscoelasticity, ferroelectric polymer composites exhibit behaviors such as creep, stress relaxation, and dynamic viscoelasticity [6], while in terms of dielectricity, they mainly exhibit dielectric relaxation phenomena [7].

Naturally, the non-linear behaviors of ferroelectric polymer composites are caused by the structural relaxation of polymer matrixes associated with molecular motions leading to a new structural equilibrium with low energy content [8]. These non-linear mechanical and electrical properties covering the time and frequency domains often cause challenges for modeling with traditional calculus. In the last several decades, fractional calculus has been regarded as an excellent mathematical tool to describe non-linear behaviors. Fractional differential operators have a long-term history dependence or memory effects, which makes them effective for modeling non-linear behaviors [9]. This has already been successfully applied in the areas of viscoelasticity [10,11], dielectric relaxation [12], anomalous diffusion [13], system control [14], and bioengineering issues [15].

In the field of ferroelectric polymer composites, Ducharne, Newell and Sebald [16] used a fractional derivative operator to simulate ferroelectric materials by explaining their dynamic behaviors as a complex combination of diffusive and dissipative behaviors. Kartci et al. [17] presented a novel analytical approach of series- and parallel-connected arbitrary-order fractional capacitors, by which three types of fabricated ferroelectric polymer and rGO-percolated P (VDF-TrFE-CFE) composite structures were fabricated and characterized. Agambayev et al. [18] developed a new fractional order capacitor where the insulator is made of a multi-walled carbon nanotube filled with PVDF-TrFE-CFE composites. However, up to now, only a few researchers have considered fractional calculus as an alliterative approach to modeling the viscoelasticity and dielectricity of ferroelectric polymer composites.

In the literature, an interesting topic is to find the correlation between viscoelastic and dielectric responses [19], both of which can be regarded as intermediate states. The mechanical characterization of viscoelasticity lies between pure elasticity and viscosity, which are the zero- and first-order derivatives of the strain rate. Similarly, dielectricity can be electrically characterized in the middle of the properties of capacitance and resistance, which are the zero- and first-order derivatives of electrical quantities. It is not difficult to imagine that if a mechanical or electrical element of fractional order is used, it is possible to characterize the viscoelasticity and dielectricity in the intermediate state. As a result, the motivation of this paper is to introduce the fractional order mechanical and electrical elements, namely the “dash-pot” and “cap-resistor”, respectively, to capture the non-linear viscoelastic and dielectric behaviors of ferroelectric polymer composites. Specifically, the effectiveness of the fractional approach in describing the viscoelastic deformation as well as the dielectric relaxation will be analyzed in detail.

In the following sections of this paper, Section 2 will first introduce the fractional order viscoelastic and dielectric elements, “dash-pot” and “cap-resistor”. Second, the mechanical and electrical experimental data of representative ferroelectric polymer composites will be selected from litearture, considering the conditions of viscoelastic deformation and dielectric relaxation. The fractional models that can describe the viscoelasticity and dielectricity of ferroelectric polymer composites will be further formulated in Section 4. and then be validated through comparisons with experimental data. Finally, conclusions will be drawn in Section 5.

## 2. The Fractional Viscoelastic and Dielectric Elements

Classical models using traditional calculus usually regard non-linear behavior as a combination of analogous elements: springs and dashpots to describe mechanical properties, and capacitors and resistors for electrical properties. Using fractional calculus, the intermediate elements were developed in the last few decades.

**Figure 1:**
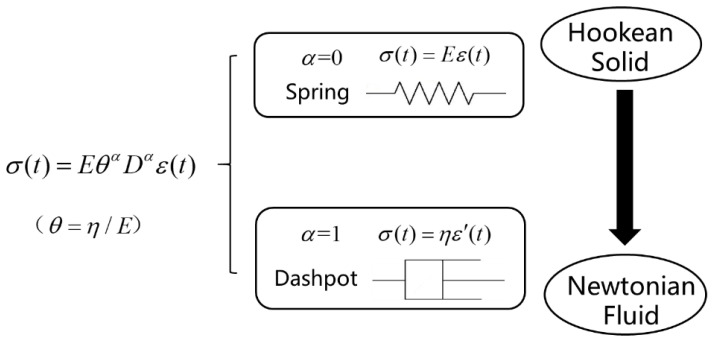
The “spring-pot” element.

For modeling the viscoelastic behavior, Smit and Vries [20] proposed the well-known fractional order constitutive equation of a “spring-pot” element:

(1) $$\sigma \left(t\right)=E\theta^{\alpha }D^{\alpha }\varepsilon \left(t\right)=E{\left(\frac{\eta }{E}\right)}^{\alpha }D^{\alpha }\varepsilon \left(t\right)\;\;\;\;\;\;\;\;0\leq \alpha <1$$

where σ and ε represent the stress and the strain, respectively, *E* is the elastic modulus, η is the viscosity and θ=η/E is the relaxation time. It can be seen from Figure 1 that when the fractional order α equals 0, Equation (1) represents Hooke’s law of an ideal solid $\sigma \left(t\right)=ED_{t}^{0}\varepsilon \left(t\right)=E\varepsilon \left(t\right)$, and Newton’s law of an ideal fluid $\sigma \left(t\right)=\eta D_{t}^{1}\varepsilon \left(t\right)=\eta \varepsilon^{\prime }\left(t\right)$ will be obtained when α=1. That is to say, Equation (1) can describe the viscoelastic behavior of intermediate materials between ideal solids and ideal fluids when the fractional order is a constant between 0 and 1.

On the other hand, Jacquelin [21] first proposed a phasance concept using the fractional derivative to associate the three fundamental electrical components of inductance, resistance, and capacitance. Reyes-Melo et al. [22] later named the electrical-fractional element “cap-resistor” to describe the dielectric relaxation phenomenon. By introducing the fractional order derivative, the fractional differential equation relating the voltage and electrical current is established:

(2) $$V\left(t\right)=\frac{\tau^{\beta }}{C}D^{\beta }Q\left(t\right)=\frac{{\left(RC\right)}^{\beta }}{C}D^{\beta }Q\left(t\right)\;\;\;\;\;\;\;\;0\leq \beta <1$$

**Figure 2:**
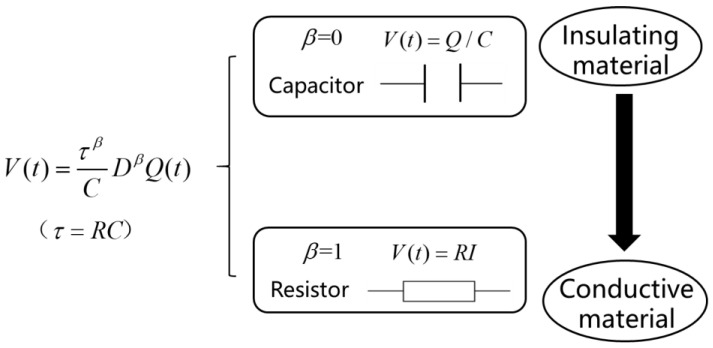
The “cap-resistor” element.

In Equation (2), *V* is the voltage, *Q* is the electric charge, *C* is the electric capacitance, *R* is the electric resistance, and τ=RC is the electric relaxation time. Similarly, as shown in Figure 2, when the fractional order β equals 0, Equation (2) is equivalent to a capacitor $V\left(t\right)=\frac{1}{C}D^{0}Q\left(t\right)=\frac{Q}{C}$, and when β=1, it can be written as a resistor $V\left(t\right)=\frac{RC}{C}Q^{\prime }\left(t\right)=RI$. Therefore, when the fractional order varies between 0 and 1, Equation (2) can represent the intermediate properties between insulating materials and conductive materials.

In theory, since the mechanical as well as electrical manifestations of ferroelectric polymer composites behave as intermediate states, their non-linear viscoelastic and dielectric properties can be captured with the aid of fractional “spring-pot” and “cap-resistor” elements. In the following sections, the fractional models that can describe the viscoelasticity and dielectricity of ferroelectric polymer composites will be further developed, and the effects of models will be validated by comparisons with experimental data of representative ferroelectric polymer composites.

## 3. Experiment

The fractional order “spring-pot” and “cap-resistor” elements are used for modeling the viscoelasticity and dielectricity, respectively. Therefore, representative data of ferroelectric polymer composites obtained by mechanical and electrical experiments were selected form the literature for validation.

In the case of viscoelasticity, the deformation of ferroelectric polymer composites in use is an important point of concern. The deformation behavior for a BaTiO_3_/P (VDF-TrFE) ferroelectric composite film under uniaxial tension was studied by Fang et al. [6]. The tensile tests were carried out at a constant crosshead speed of 0.2 mm/min. The stress–strain curve for BaTiO_3_/P (VDF-TrFE) ferroelectric composite film can be seen in the Figure 1 of reference [6]. It is shown that due to the viscoelasticity of the polymeric materials, the stress–strain response depends on the strain rate, which exhibits typical viscoelastic behavior.

On the other hand, the characterization of the dielectricity of ferroelectric polymer composites focuses on the dielectric relaxation, which can be characterized by their dielectric constants and dielectric losses. Therefore, the dielectric properties of PVDF-HFP/NKBT (Na_0.25_K_0.25_Bi_0.5_TiO_3_) composites experimentally studied by Pavlović et al. [2] were utilized for model validation. The dielectric constant and dielectric loss curves of PVDF-HFP/NKBT composites as a function of frequency are presented in the Figure 4 of Reference [2], in which the green line referring to the composites with 10 wt.% filler is utilized for analysis. The dielectric constant of PVDF-HFP/NKBT composites generally decreases with increasing frequency from 0.1 to 10^6^ Hz. A decrease is especially pronounced in the region of frequencies lower than 10 Hz due to polarization effects [23]. Electrode polarization is a consequence of the accumulation of the charges at the surface of the electrode while under the force of the electric field, which will cause the dielectric constant as well as the dielectric loss at low frequencies to be much larger then at high frequencies. Similarly, it can be found in the dielectric loss curve that the dielectric loss of PVDF-HFP/NKBT composites decreases with the increase of frequency up to 10^4^ Hz and the value of dielectric loss is relatively high at low frequencies under 10 Hz due to polarization effects. However, at frequencies above 10^4^ Hz another process is observed, which is usually associated with dipole relaxations [23], that the activation of the molecular dipoles in polymer matrix will result in an increase of dielectric loss. As a result, the dielectric loss of PVDF-HFP/NKBT composites starts to increase with frequency increases from 10^4^ to 10^6^ Hz.

## 4. Results and Discussion

The fractional derivative operators can be solved in both the time and frequency domains, which makes it flexible to meet the requirements in the different cases of experimental data for both viscoelasticity and dielectricity.

### 4.1. Viscoelasticity

To use the “spring-pot” element to model the stress–strain behavior of a BaTiO_3_/P (VDF-TrFE) ferroelectric composite film, the Riemann–Liouville definition of a fractional derivative was selected, which is defined as:

(3) $$D_{t}^{\alpha }f\left(t\right)=\frac{1}{\Gamma \left(1-\alpha \right)}\frac{d}{dt}\int_{\alpha }^{t}\frac{f\left(\tau \right)d\tau }{{\left(t-\tau \right)}^{\alpha }}\;\;\;\;\;\;\;0\leq \alpha <1$$

where Γ(*) is the Gamma function:

(4) $$\Gamma \left(t\right)=\int_{0}^{t}e^{-t}\tau^{t-1}d\tau$$

On the basis of Riemann–Liouville definition, when the strain rate is a constant function ε(t)=ct, Equation (1) can be written into:

(5) $$\sigma \left(t\right)=E\theta^{\alpha }\frac{ct^{1-\alpha }}{\Gamma \left(2-\alpha \right)}$$

Finally, the fractional order viscoelastic model of the stress–strain relationship can be obtained:

(6) $$\sigma \left(t\right)=E{\left(c\theta \right)}^{\alpha }\frac{\varepsilon {\left(t\right)}^{1-\alpha }}{\Gamma \left(2-\alpha \right)}$$

**Figure 3:**
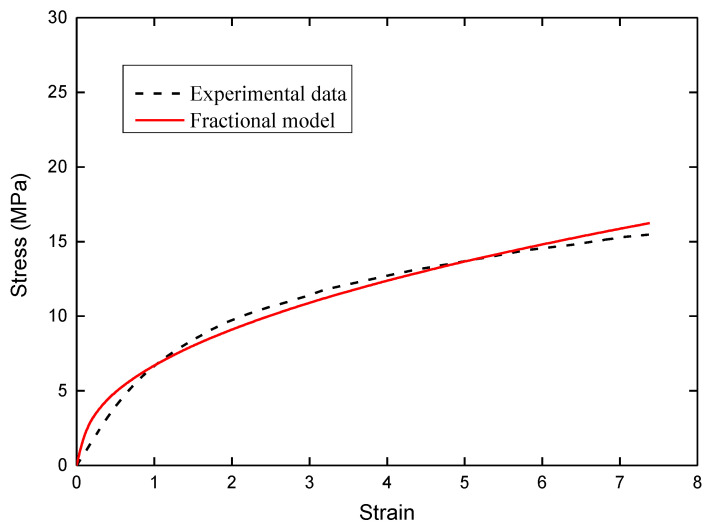
Comparison between the fractional order viscoelastic model and the uniaxial tension data of a BaTiO_3_/P (VDF-TrFE) ferroelectric composite film.

The method of least squares was employed to fit Equation (6) to the uniaxial tension data of a BaTiO_3_/P (VDF-TrFE) ferroelectric composite film. During data fitting, the model parameters were obtained as follows: *E* is 701 MPa, θ is 3.465 s, and the fractional order α is equal to 0.558. As a result, the comparison between the fractional order viscoelastic model and the experimental data is shown in Figure 3. It is clearly demonstrated that the stress–strain curve obtained by the fractional viscoelastic model agrees well with the uniaxial tension data of a BaTiO_3_/P (VDF-TrFE) ferroelectric composite film.

### 4.2. Dielectricity

The dielectric constant and dielectric loss of ferroelectric polymer composites are the real part and imaginary part of their complex permittivity, respectively. Therefore, the “cap-resistor” element needs to be further formulated into the frequency domain to obtain the fractional order dielectric model.

The Fourier transform of a fractional derivative operator of order β is:

(7) $$F\left\{\frac{d^{\beta }f(t)}{dt^{\beta }},\omega \right\}={\left(i\omega \right)}^{\beta }F(\omega )$$

Then the Fourier transform of Equation (2) leads to:

(8) $$\tilde{V}(\omega )=\frac{{\left(i\omega \tau \right)}^{\beta }}{C}\tilde{Q}(\omega )$$

The complex capacitance of a single “cap-resistor” can be calculated:

(9) $$\tilde{C}(\omega )=\frac{C}{{\left(i\omega \tau \right)}^{\beta }}$$

The corresponding complex permittivity, dielectric constant, and dielectric loss are:

(10) $$\tilde{\varepsilon }(\omega )=\frac{\varepsilon }{{\left(i\omega \tau \right)}^{\beta }}$$

(11) $$\varepsilon^{\prime }(\omega )=\varepsilon {\left(\omega \tau \right)}^{-\beta }\cos(\pi \beta /2)$$

(12) $$\varepsilon^{\prime \prime }(\omega )=\varepsilon {\left(\omega \tau \right)}^{-\beta }\sin(\pi \beta /2)$$

where ε reflects the dielectric constant of free space.

In theory, the fractional order dielectric model of Equations (11) and (12) can capture the dielectric constant and the dielectric loss curves of PVDF-HFP/NKBT composites. The same method of least squares was employed for data fitting and to determine the model parameters. The values of root mean squared error (RMSE) and coefficient of determination (R-square) were also calculated to evaluate the fitting results of the fractional order dielectric model. It is to be noted that due to polarization effects, the frequency dependences of PVDF-HFP/NKBT composites are different at low frequencies and high frequencies. This results in changes in material parameters and model constants before and after 10 Hz. Therefore, the fitting procedures were separated into two regions with different values of model parameters.

**Figure 4:**
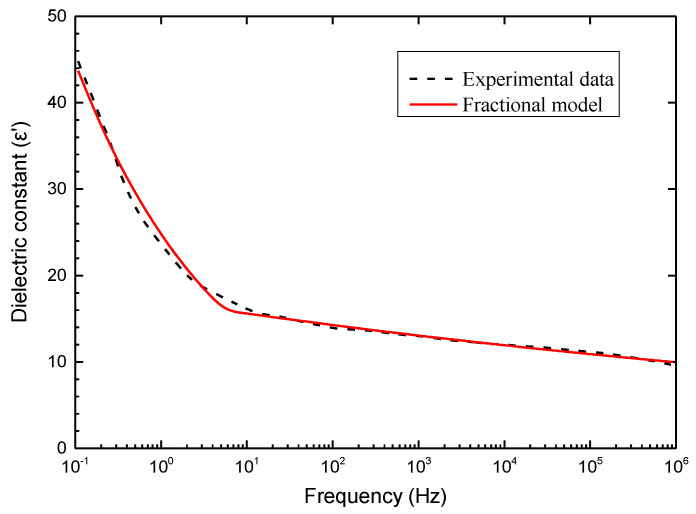
Comparison between the fractional order dielectric model and the dielectric constant of PVDF-HFP/NKBT composites.

The comparison between the fractional order dielectric model and the dielectric constant of PVDF-HFP/NKBT composites is plotted in Figure 4. The two sets of model parameters as well as evaluations before and after 10 Hz are given in Table 1. As can be seen in Figure 4, the results of the fractional order dielectric model are in good agreement with the dielectric constant curve in both the low and high frequency regions.

**Figure 5:**
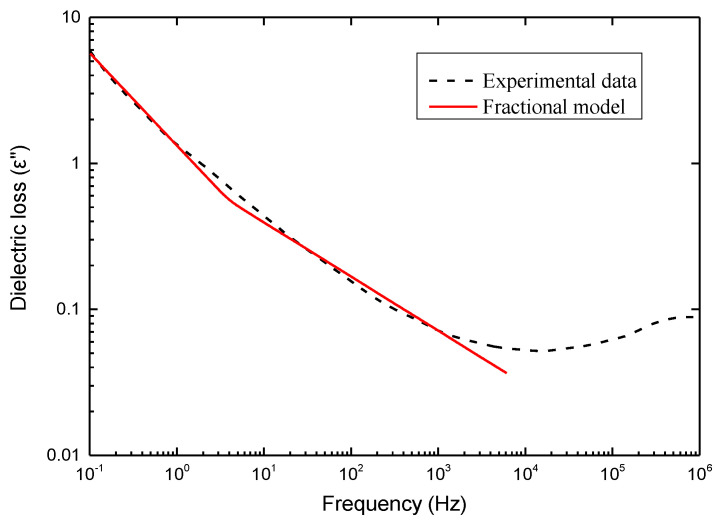
Comparison between the fractional order dielectric model and the dielectric loss of PVDF-HFP/NKBT composites.

The model fitting result of dielectric loss of PVDF-HFP/NKBT composites is shown in Figure 5. The model parameters and evaluations for different frequency regions are listed in Table 2. Likewise, two different sets of parameters were used in the fractional order dielectric model for the low frequency region of 0.1 to 10 Hz and high frequency region of 10 to 10^4^ Hz. It is demonstrated by Figure 5 that the modeling results satisfy the dielectric loss curve at frequencies smaller than 10^4^ Hz. However, as has been explained in Section 3, when the frequency is higher than 10^4^ Hz the dielectric loss curve of PVDF-HFP/NKBT composites starts to rise due to dipole relaxation. This phenomenon causes the fractional order dielectric model to fail to describe the dielectric loss curve with a range greater than 10^4^ Hz.

In summary, the fractional order dielectric model can effectively reveal the frequency dependence as well as polarization effect of the dielectric constant and dielectric loss of ferroelectric polymer composites. Only when the dipole relaxations occur at higher frequencies can the proposed model not describe the rise of the dielectric loss curve.

## 5. Conclusions

In this paper, the fractional order calculus approach has been introduced to describe the non-linear mechanical and electric behavior of ferroelectric polymer composites originating from the viscoelastic and dielectric relaxation of polymer matrixes. The fractional elements for viscoelasticity and dielectricity are “spring-pot” and “cap-resistor”, which can capture the intermediate properties between spring and dashpot or capacitor and resistor, respectively.

In case of viscoelasticity, the “spring-pot” equation was directly used to model the uniaxial tension behavior of a BT/P (VDF-TrFE) ferroelectric composite film. It was found that the fractional viscoelastic model agrees well with the experimental data. For modeling the dielectricity of ferroelectric polymer composites, which is usually characterized by dielectric constants and dielectric losses, the “cap-resistor” element was further formulated into the frequency domain to obtain the fractional order dielectric model. The experimental results of PVDF-HFP/NKBT composites were adopted to validate the fractional order dielectric model. The fitting procedures were separated into low and high frequency regions with two sets of model parameters due to the polarization effect. It was demonstrated that the fractional order dielectric model can effectively reveal the frequency dependence of the dielectric constant and dielectric loss of ferroelectric polymer composites. Only when the dipole relaxations occur at higher frequencies can the proposed model not describe the rise of the dielectric loss curve.

## Figures and Tables

**Table 1:** Model parameters and evaluations for dielectric constant.

*w* (Hz)	ε	τ	β	RMSE	R^2^
0.1–10	23.94	0.64	0.26	1.49	0.98
10–10^6^	16.99	0.83	0.04	0.23	0.98

**Table 2:** Model parameters and evaluations for dielectric loss.

*w* (Hz)	ε	τ	β	RMSE	R^2^
0.1–10	7.02	10.53	0.63	0.16	0.99
10–10^4^	1.65	0.95	0.37	0.01	0.98
